# Risk for High Depressive Symptoms in Diagnosed and Previously Undetected Diabetes: 5-Year Follow-Up Results of the Heinz Nixdorf Recall Study

**DOI:** 10.1371/journal.pone.0056300

**Published:** 2013-02-18

**Authors:** Andrea Icks, Bernd Albers, Burkhard Haastert, Sonali Pechlivanis, Noreen Pundt, Uta Slomiany, Raimund Erbel, Karl-Heinz Jöckel, Johannes Kruse, Bernd Kulzer, Bettina Nowotny, Christian Herder, Guido Giani, Susanne Moebus

**Affiliations:** 1 Institute of Biometrics and Epidemiology, German Diabetes Center, Leibniz Center for Diabetes Research at the Heinrich Heine University Düsseldorf, Düsseldorf, Germany; 2 Department of Public Health, Faculty of Medicine, Center of Health and Society, Heinrich Heine University Düsseldorf, Düsseldorf, Germany; 3. mediStatistica, Neuenrade, Germany; 4 Institute for Medical Informatics, Biometry and Epidemiology, University Hospital of Essen, Essen, Germany; 5 Clinic for Cardiology, University Hospital of Essen, Essen, Germany; 6 Clinic for Psychosomatics and Psychotherapy, University Hospital Gießen/Marburg, Gießen, Germany; 7 Research Institute of the Diabetes Academy Mergentheim and Diabetes Centre Mergentheim, Bad Mergentheim, Germany; 8 Institute for Clinical Diabetology, German Diabetes Center, Leibniz Center for Diabetes Research at Heinrich Heine University Düsseldorf, Düsseldorf, Germany; Tehran University of Medical Sciences, Islamic Republic of Iran

## Abstract

**Objective:**

The objective of this study was to determine the risk for the development of high depressive symptoms in study participants with diagnosed and previously undetected diabetes mellitus compared to those without diabetes in a prospective population-based cohort study in Germany.

**Methods:**

We estimated the 5-year cumulative incidence of high depressive symptoms in participants without high depressive symptoms at baseline (n = 3,633, 51.4% men, mean age (SD) 59.1 (7.6) years, 7.0% diagnosed diabetes, 5.3% previously undetected diabetes) from the population-based Heinz Nixdorf Recall study. Diabetes was assessed by self-report, medication, and blood glucose. High depressive symptoms were assessed using CES-D. We calculated odds ratios and their corresponding 95% confidence interval, using multiple logistic regression analyses.

**Result:**

Cumulative 5-year incidences (95% CI) of high depressive symptoms in participants with diagnosed, undetected, and without diabetes were 7.1 (4.2–10.9), 4.1 (1.8–8.0), and 6.5 (5.6–7.4), respectively. The age-sex-adjusted OR for developing high depressive symptoms was 1.22 (0.74–2.03) in participants with diagnosed compared to those without diabetes, and 1.00 (0.59–1.68) after adjustment for BMI, physical activity, education, stroke, and myocardial infarction. The age-sex adjusted OR for developing high depressive symptoms in participants with previously undetected diabetes compared to those without diabetes was 0.72; 0.35–1.48; and fully adjusted 0.62; 0.30–1.30.

**Conclusion:**

We found no significant associations, maybe due to low power. However, our results are in line with a recent meta-analysis suggesting that risk of developing high depressive symptoms in patients with diagnosed diabetes may be moderately higher than in those without diabetes, and that comorbidity may explain in part this association. In participants with previously undetected diabetes, this first longitudinal study indicates that the risk is not increased or may even be decreased. These results support the hypothesis that high depressive symptoms develop due to diabetes-related burden and comorbidity and not due to hyperglycemia or hyperinsulinaemia.

## Introduction

There is sufficient evidence for an association between prevalent depressive disorders and diabetes, with up to twofold higher prevalence of depressive disorders in persons with diabetes [1]. The combination of diabetes and depressive disorders is of high clinical relevance, due to an increased risk of mortality [2]–[4], micro- and macro-vascular co-morbidities [5], and physical and psychological disabilities [3], [6], [7] in people with a combination of these two health problems.

It remains controversial whether diabetes predicts depressive disorders or vice versa or whether there is a bidirectional association. Prospective studies examining the association between diabetes and high depressive symptoms are scarce and reveal conflicting results. A meta-analysis including seven studies reported a weakly increased risk for the development of high depressive symptoms in individuals with diabetes compared with those without diabetes (RR = 1.15, 95% CI 1.02–1.30) [8]. A more recent meta-analysis found the relative risk to be slightly higher (RR 1.24; 95% CI 1.09–1.40) [9].

Possible reasons for a higher risk of high depressive symptoms in diabetes are still a matter of debate [8], [9], [10]. Depression may develop in association with somatic disorders as (diabetes-related) comorbidity. However, also hyperglycemia or hyperinsulinemia per se is being discussed as a possible underlying factor for an increased risk of high depressive symptoms as follows: Biochemical changes associated with diabetes increase the activity of the hypothalamic–pituitary–adrenal axis, which may in turn induce depression [8]. If this explanation were true, one would expect not only increased high depressive symptoms in diagnosed diabetes but also in previously undetected diabetes. However, only a few cross-sectional studies have investigated high depressive symptoms in participants with previously undetected diabetes. A recent systematic review and meta-analysis of Nouwen et al. including 10 cross-sectional studies has found no difference between the prevalence of high depressive symptoms in individuals with undetected diabetes and without diabetes (OR 0.94; 95% CI 0.71–1.25) [11]. The analysis of the cross-sectional baseline data of the population-based Heinz Nixdorf Recall study yielded an even lower prevalence of high depressive symptoms in men with previously undetected diabetes than in those without diabetes [12]. To the best of our knowledge, no study to date has investigated the association between previously undetected diabetes and the onset of high depressive symptoms in a longitudinal setting.

In light of these findings, we sought to estimate the 5-year cumulative incidence of high depressive symptoms in participants with diagnosed and previously undetected diabetes and to compare it with that in participants without diabetes in a population-based sample. By calculating the odds ratios, we estimated the effects of diabetes and undetected diabetes on the development of high depressive symptoms.

## Methods

### Population

We analysed baseline and follow-up data from the ongoing population-based prospective Heinz Nixdorf Recall study, performed in large adjacent cities (Essen, Mülheim, Bochum) of the Ruhr Area in Germany. The design of the study has been described elsewhere in detail [13]. Briefly, the cohort comprises 4,814 randomly selected men and women aged 45–75 years at baseline. The baseline visits were performed at the Epidemiological Study Centre, Essen, between 2000 and 2003, the 5-year follow-up visits between 2005 and 2008, with a median follow-up of 5.1 years. The baseline recruitment efficacy was 55.8% [14] and the 5-years follow up response 90.2% [15]. Data assessment at baseline and 5-year follow-up included self-administered questionnaires, face-to-face interviews for personal risk factor assessment and various medical conditions, conducted by trained study personnel. Comprehensive laboratory tests, anthropometric and blood pressure measurements were performed according to standard study protocols. The study was approved by the relevant institutional ethics committees and followed strict internal and external quality assurance protocols. All participants gave their written informed consents.

The effective sample size in the study was 4,157 participants. For the present analysis, we excluded 373 participants with high depressive symptoms at baseline (for definition see below), 119 with unknown depressive symptoms at baseline and 32 participants with unknown status of depressive symptoms at follow-up, yielding the final study sample of n = 3,633 participants.

### Variables

#### Outcome: high depressive symptoms

We assessed high depressive symptoms using the 15-item short form of the Center for Epidemiologic Studies Depression scale (CES-D), a commonly used instrument [16], [17] and assumed a cut-off point of ≥17, as defined in validation studies [18]. The short form of the CES-D has been validated in detail [19], [20]. It identifies patients with clinical depression when compared to ICD 10– diagnoses with a sensitivity of 91.8 and a specifity of 83.9 [21].

#### Exposure assessment: diagnosed and previously undetected diabetes

Diagnosed diabetes mellitus was defined if clinician-documented diagnosis of diabetes was reported in the medical history interview or if glucose-lowering drugs were taken [12]. Previously undetected diabetes was considered when (i) fasting glucose was ≥7.0 mmol/l (60% and 79% of study participants were in a fasting state at baseline and follow-up, respectively, with fasting state defined as fasting for ≥8 hours) or random blood glucose ≥11.1 mmol/l [22], [23] (the remaining participants were in a non-fasting state) and (ii) participants had not reported diabetes diagnosis and were not taking any glucose-lowering agents. In both baseline and follow-up examinations, the participants were informed when previously undetected diabetes was identified, and suggested to contact their general practitioners.

#### Covariates

Covariates known to modify depressive symptoms (outcome), that were also associated with diabetes (exposure of interest), were identified from clinical experience and the literature and discussed prior to the analyses. At baseline, physical exercise was assessed by asking our participants about the kind and duration of exercise performed in the preceding month, whereby ‘physically inactive’ meant having performed no physical exercise at all [24]. Education was used as a proxy for the socio-economic status, classified in years of formal education according to the “International Standard Classification of Education” [25] combining school and vocational training: ≤10 years (lowest education), 11–13 years, 14–17 years and ≥18 years (highest education). Information on previous medical events including stroke and myocardial infarction were assessed during a standardized medical computer-assisted personal interview. Height and weight were measured by clinical examination according to standard protocols.

### Statistical Analysis

The baseline characteristics, stratified by diabetes status, were summarized by using descriptive statistics. Five-year cumulative incidences of high depressive symptoms were estimated as proportions of participants developing high depressive symptoms during follow-up. We estimated the cumulative incidences with 95% confidence intervals (95%-CI) in the population of participants with diagnosed, with previously undetected, and without diabetes at baseline.

The association between diabetes (diagnosed or previously undetected) at baseline and the risk of developing high depressive symptoms during follow-up was analysed by using multiple logistic regression models, with high depressive symptoms at follow-up as the dependent variable, and baseline diabetes (diagnosed and undetected diabetes separately on both corresponding subpopulations of non-diabetic participants and diagnosed resp. undetected diabetic cases) and additional covariates as independent variables. Model 1 was adjusted only for age and sex. In Model 2, we included the further covariates (education, physical activity, BMI, stroke, and myocardial infarction).

### Sensitivity Analysis

The aforementioned meta-analysis [11] reported higher relative risks for developing depressive symptoms in individuals with compared to those without diabetes when diagnostic-based instead of questionnaire-based criteria were used. Furthermore, excluding individuals with depression history at baseline revealed lower relative risks [9]. Thus, we performed corresponding sensitivity analyses: (1) We defined high depression symptoms using the questionnaire-based CES-D and additionally a physician-documented life-time depression diagnosis in the medical history. (2) We excluded participants with a physician-documented life-time depression diagnosis in the medical history from the analysis. Furthermore, considering that the prevalence of subclinical depression as a strong predictor of depression may differ between participants with diagnosed or undetected diabetes and those with normal glucose tolerance, we adjusted for this condition by adding an indicator as indendent variable in the logistic regression models. Since no corresponding cut-off for the CES-D is defined, we assumed subclinical depression when CES-D-values were between mean+standard deviation ( = 7.9+6.1 on the whole population, n = 4,038, 119 missings) and the cut-off for clinical depression, that is between 14 and 17. Additionally, in an explorative analysis we estimated the association between comorbidity (stroke, myocardial infarction, diabetic retinopathy, chronic kidney disease, or diabetic foot syndrome) and development of depression in participants with diagnosed diabetes.

## Results

### Baseline Characteristics of the Study Cohort

The baseline characteristics of our study cohort stratified by diabetes status are presented in Table 1. A total of 51.4% of the study population were men and the mean age (SD) was 59.1 years (7.6). As expected, participants with diagnosed and previously undetected diabetes were older, more frequently male, were less educated, had a higher BMI, and were less often physically active than participants without diabetes. Furthermore, they reported more often a history of myocardial infarction or stroke.

**Table 1:** Baseline participant characteristics, stratified by diabetes status.

	Diagnosed Diabetes (n = 255)	Undetected Diabetes(n = 194)	No diabetes (n = 3,184)
Age, mean (SD), y	61.6 (7.2)	60.5 (7.4)	58.9 (7.6)
Male (%)	63.9	70.6	49.2
Education: years of school % (95% CI) (3 missings)			
≤10	12.2 (8.4–16.8)	8.8 (5.2–13.7)	8.2 (7.3–9.2)
11–13	54.5 (48.2–60.7)	56.2 (48.9–63.3)	56.2 (54.4–57.9)
14–17	23.9 (18.8–29.6)	26.8 (20.7–33.6)	23.0 (21.6–24.5)
≥18 years	9.4 (6.1–13.7)	8.2 (4.8–13.0)	12.6 (11.4–13.8)
No regular physical exercise % (95% CI)	54.5 (48.2–60.7)	56.2 (48.9–63.3)	41.2 (39.5–42.9)
BMI >30 kg/m^2^ (95% CI) (15 missings)	43.3 (37.1–49.6)	49.7 (42.5–57.0)	21.8 (20.4–23.3)
Co-Morbidities % (95% CI)^1^			
CVD (8 missings)	13.3 (9.4–18.1)	10.9 (6.9–16.2)	4.6 (3.9–5.4)
Myocardial infarction (32 missings)	7.5 (4.6–11.5)	8.5 (4.9–13.4)	3.3 (2.7–3.9)
Stroke (19 missings)	6.7 (3.9–10.5)	1.0 (0.1–3.7)	1.8 (1.4–2.4)
Diabetes-related co-morbidities % (95% CI)			
Retinopathy (19 missings)	12.3 (8.4–17.2)		
Blindness (13 missings)	0.8 (0.1–3.0)		
Proteinuria (67 missings)	17.6 (12.4–23.8)		
Renal failure (13 missings)	2.1 (0.7–4.8)		
Renal replacement therapy (13 missings)	0.8 (0.1–3.0)		
Neuropathy (18 missings)	31.6 (25.8–38.0)		
Amputation (13 missings)	1.7 (0.5–4.2)		

1 at baseline: ever diagnosed by a physician.

### Cumulative Incidences and Odds Ratios from Multiple Analysis

During follow-up (mean 5.1±0.3 years), we observed a total of 232 incident cases of high depressive symptoms in n = 18, 8, and 206 participants with diagnosed, with previously undetected, and without diabetes, respectively. The 5-year cumulative incidences were 7.1% (95%CI 4.2–10.9) in those with diagnosed diabetes, 4.1% (1.8–8.0) in those with undetected diabetes, and 6.5% (5.6–7.4) in participants without diabetes.

In the multiple regression analysis, the age- and sex-adjusted risk for developing high depressive symptoms in participants with diagnosed diabetes was 1.22 (95%CI, 0.74–2.03) compared to those with no diabetes (Table 2). After adjustment for further covariates the OR was 1.00 (0.59–1.68). On the other hand, the age- and sex-adjusted OR for developing high depressive in participants with previously undetected diabetes compared to those without diabetes was 0.72 (0.35–1.48), and 0.62 (0.30–1.30) after full adjustment (Table 2).

**Table 2:** Multiple logistic regression models analyzing the relationship between diagnosed and undetected diabetes and the development of high depressive symptoms.

Model/Variable	Diagnosed diabetes^1^	Undetected diabetes^1^
	Odds Ratios (95% CI)	Odds Ratios (95% CI)
**Model 1**	(all : n = 3,439)	(all: n = 3,378)
Diabetes (diagnosed/undetected)	1.22 (0.74–2.03)	0.72 (0.35–1.48)
Age (per year)	1.00 (0.98–1.01)	1.00 (0.98–1.01)
Sex (male)	0.53 (0.40–0.71)	0.54 (0.40–0.72)
**Model 2**	(n = 3,422)	(n = 3,361)
Diabetes (diagnosed/undetected)	1.00 (0.59–1.68)	0.62 (0.30–1.30)
Age (per year)	0.99 (0.97–1.004)	0.99 (0.97–1.01)
Sex (male)BMI (>30 versus ≤30 kg/m^2^)	0.58 (0.43–0.78)1.24 (0.91–1.69)	0.58 (0.43–0.79)1.27 (0.92–1.74)
Myocardial infarction (yes versus no)^2^	1.18 (0.56–2.50)	1.27 (0.60–2.70)
Stroke (yes versus no) ^2^	3.24 (1.72–6.11)	3.07 (1.51–6.25)
No regular physical activity (versus yes)	1.49 (1.13–1.97)	1.54 (1.16–2.05)
Education (ordinal changes per category step 1–4)	0.77 (0.63–0.94)	0.78 (0.64–0.95)

1 Separate logistic regression models on different subpopulations.

^2^Baseline assessment: ever diagnosed by a physician, few missings (19 resp. 32 overall, cf. table 1) defined as “no”.

### Sensitivity Analyses

The exclusion of participants with a depression in the medical history at baseline (n = 242) did not change the results substantially (diagnosed diabetes versus no diabetes: age-sex adjusted OR 1.34 (0.79–2.26; n = 3,206); previously undetected diabetes versus no diabetes: age-sex-adjusted OR 0.84 (0.41–1.75; n = 3,145).

When we defined high depressive symptoms using CES-D and additionally a physician-documented depression in the medical history, the age- and sex-adjusted OR for the development of high depressive symptoms in participants with diabetes was 1.57 (0.96–2.57; n = 2,571) compared with participants with no diabetes, and 1.33 (0.80–2.22, n = 2,557) after controlling for further covariates. In participants with previously undetected diabetes, we achieved almost unchanged results as in the base case analysis (age-sex-adjusted OR 0.74 (0.36–1.54; n = 2,538; fully adjusted model 0.65 (0.31–1.37, n = 2,524).

Subclinical depression was significantly and strongly associated with the risk for developing high depressive symptoms (OR about 6). However, the base case ORs remained almost unchanged in all models (data not shown).

With regard to comorbidity in participants with diagnosed diabetes, the age-sex-adjusted OR for developing high depressive symptoms in participants with comorbidity (n = 255) compared to those without comorbidity (n = 217) was 1.55 (95% CI 0.53–4.53), and 1.48 (0.49–4.45) after full adjustment.

## Discussion

### Study Findings and Implications

Our 5-year observational study is, to the best of our knowledge, the first study that analysed the incidence of high depressive symptoms in individuals with diagnosed as well as with previously undiagnosed diabetes mellitus versus participants without diabetes in a prospective study design.

We did not find a significant association between diagnosed diabetes and the risk to develop high depressive symptoms. However, an age-sex-adjusted risk increase of about 20% as in our study would be well in line with a recently published meta-analysis including 11 studies with 48,800 study participants which found an OR of 1.24 (95% CI 1.09–1.40) [9]. Consistent with the findings from de Jonge [26] the OR tended to diminish after controlling for covariates like comorbidities. This finding supports the hypothesis that not the metabolic situation per se, but diabetes-associated comorbidities increase the risk of developing high depressive symptoms. In our regression model we observed that particularly stroke is associated with an increased risk for high depressive symptoms, which is in line with earlier findings [27]. Also physical inactivity was associated with an increased risk for high depressive symptoms. Furthermore, the risk for high depressive tended to be increased with obesity. This is in line with a recent review showing that obesity can induce low self-esteem and body dissatisfaction and increases the risk for developing a depression [28]. An association between diabetes-associated comorbidities and the risk of developing high depressive symptoms has been observed in previous studies [29], and is also supported by our explorative analysis of participants with diagnosed diabetes: those with comorbidities tended to have a higher risk of developing high depression symptoms compared to those without comorbidities. However, the results are yet uncertain due to wide confidence intervals.

No significant association between undetected diabetes and the risk of developing high depressive symptoms was found. Our study results may indicate that the risk of high depressive symptoms in participants with previously undetected diabetes at baseline could be even lower compared to participants without diabetes. The observation that there is no association between undetected diabetes and high depressive symptoms has been described: In a review of cross-sectional studies, the prevalence odds ratio of high depressive symptoms in participants with undetected diabetes and those without diabetes was 0.94 (0.71–1.25) [11]. Note that in our study, participants without previously detected diabetes have been informed about their elevated blood glucose measures and recommended to consult their doctors. Hence, they have not longer been undetected during the follow-up period. The question arises why these participants have still a lower risk for developing high depressive symptoms than those with previously diagnosed diabetes. This may be explained by the hypothesis that depressive symptoms are associated with burden of treatment and the presence of diabetes-related complications, as discussed by Nouwen in its review about the prevalence of depressive symptoms in people with undetected diabetes, and confirmed e.g. by the finding that untreated diabetes has not been associated with an increased risk of developing high depressive symptoms [29]: Our follow-up investigation revealed, that only about one third of participants with undetected diabetes at baseline received antihyperglycemic medication (64 of 194), compared to 81% (206/255) of the participants with previously diagnosed diabetes. At baseline, HbA1c was 6.3±1.5% and 6.9±1.3% in participants with previously undetected diabetes and those with previously diagnosed diabetes, respectively. In the follow-up investigation, 8% (16/194) of participants with undetected diabetes at baseline had diabetes-related complications, compared to 25% (63/255) of those with previously diagnosed diabetes. Thus, indeed, the intensity of treatment and the severity of diabetes can be considered to be lower in participants with previously undetected diabetes compared to those with previously diagnosed participants. U However, further studies including a higher number of participants and additional covariates, e.g. stress, are warranted to analyse the underlying reasons.

Several sensitivity analyses did not substantially change our findings. We (i) excluded participants who reported a physician-documented lifetime history of depression, and (ii) performed all analyses defining high depressive symptoms as a CES-D score ≥17 or a lifetime history of physician-documented depression. In both sensitivity analyses, the risk of developing high depression symptoms in participants with diagnosed diabetes compared to those without diabetes tended to be about 30% and 40% increased, however, with confidence intervals including the 1. A somewhat higher risk would be well in line with the meta-analysis of Nouwen, who found higher risk ratios when depression was assessed by clinical interviews compared to questionnaire-based definition of high depressive symptoms [9]. In participants with undetected diabetes compared to those without diabetes, the decrease of risk of developing high depressive symptoms tended to be somewhat larger than in the base case analysis, however, confidence intervals were still wide.

### Study Strengths and Limitations

The strength of the study is that it is a well-performed investigation including carefully assessed variables. The number of participants lost to follow-up is very low. Since about two thirds of the participants were in a fasting state at baseline, we could identify undetected diabetes. High depressive symptoms could be assessed by a widely used and well established instrument.

However, the study has several limitations: (1) Not all participants were examined in a fasting state and no oral glucose tolerance test was performed, causing potential misclassifications. However, our study results remained unchanged when excluding non-fasting participants without diabetes from our analyses. (2) The number of participants with high depressive symptoms in the groups with diagnosed and undetected diabetes was low, resulting in wide confidence intervals and missing statistical significance. (3) The gold standard to for assessment of depression is a structured interview. However, this is difficult to realize in an epidemiological studies involving a large number of participants. Thus, we used the CES-D to assess depressive symptoms. This well-established instrument has been used in a number of studies which analysed the association between diabetes and depression [9], [30]. (4) Emotional stress as a predictor of depression was not assessed [31]. (5) We had to exclude some participants due to missing information about their diabetes status. However, the number was small, and we have no indication that this could have led to any selection bias.

### Conclusion

Although we found no significant associations, our results suggest that the risk of developing high depressive symptoms in participants with diagnosed diabetes may be moderately higher than in those without diabetes, and that co-morbidity and lifestyle may explain in part this association. For the first time we estimated the risk of developing high depressive symptoms in participants with previously undetected diabetes and found no significant association, maybe an even lower risk than in participants without diabetes. However, our results have to be replicated and corroborated in studies with a larger number of cases and higher statistical power. Corresponding results would support the hypothesis that high depressive symptoms develop mainly due to diabetes-related treatment and comorbidity.
